# Combination interventions for Hepatitis C and Cirrhosis reduction among people who inject drugs: An agent-based, networked population simulation experiment

**DOI:** 10.1371/journal.pone.0206356

**Published:** 2018-11-29

**Authors:** Bilal Khan, Ian Duncan, Mohamad Saad, Daniel Schaefer, Ashly Jordan, Daniel Smith, Alan Neaigus, Don Des Jarlais, Holly Hagan, Kirk Dombrowski

**Affiliations:** 1 Department of Sociology, University of Nebraska, Lincoln NE, United States of America; 2 Rory Meyers College of Nursing, New York University, New York, NY, United States of America; 3 Center for Drug Use and HIV Research, New York University, New York, NY, United States of America; 4 Department of Epidemiology, Mailman School of Public Health, Columbia University, New York, NY, United States of America; 5 Icahn School of Medicine at Mount Sinai, New York, NY, United States of America; University of Cincinnati College of Medicine, UNITED STATES

## Abstract

Hepatitis C virus (HCV) infection is endemic in people who inject drugs (PWID), with prevalence estimates above 60% for PWID in the United States. Previous modeling studies suggest that direct acting antiviral (DAA) treatment can lower overall prevalence in this population, but treatment is often delayed until the onset of advanced liver disease (fibrosis stage 3 or later) due to cost. Lower cost interventions featuring syringe access (SA) and medically assisted treatment (MAT) have shown mixed results in lowering HCV rates below current levels. However. little is known about the potential cumulative effects of combining DAA and MAT treatment. While simulation experiments can reveal likely long-term effects, most prior simulations have been performed on closed populations of model agents—a scenario quite different from the open, mobile populations known to most health agencies. This paper uses data from the Centers for Disease Control’s National HIV Behavioral Surveillance project, IDU round 3, collected in New York City in 2012 to parameterize simulations of open populations. To test the effect of combining DAA treatment with SA/MAT participation, multiple, scaled implementations of the two intervention strategies were simulated. Our results show that, in an open population, SA/MAT by itself has only small effects on HCV prevalence, while DAA treatment by itself can lower both HCV and HCV-related advanced liver disease prevalence. More importantly, the simulation experiments suggest that combinations of the two strategies can, when implemented together and at sufficient levels, dramatically reduce HCV incidence. We conclude that adopting SA/MAT implementations alongside DAA interventions can play a critical role in reducing the long-term consequences of ongoing HCV infection.

## Introduction

In the United States, hepatitis C virus (HCV) infection is endemic in people who inject drugs (PWID), with approximately 60% having chronic infection, and incidence of infection among new injectors varying between 15-35 per 100 person-years of observation [1]. Beginning in 2007, HCV-related deaths in the US exceeded deaths related to HIV, and currently surpass deaths from all other notifiable infections [2]. In high-income countries, HCV is the underlying causal factor for more than half of cases of hepatocellular carcinoma (HCC), and HCC incidence will continue to climb in the coming years due to advancing infections in the PWID (and formerly PWID) population. There are highly effective and tolerable treatments for chronic HCV infection, but the costs of these direct-acting antiviral medications (DAAs) are high and currently fewer than 5-10%—the estimate is imprecise—of PWID are have been treated [3].

Although mathematical modeling has shown that HCV treatment using the DAAs can be cost-effective [4, 5], currently 23 U.S. states require that patients have advanced HCV diagnosis (Metavir fibrosis stage F3 or cirrhosis) in order to approve publicly funded medical treatment [6]. In contrast, “treatment as prevention” has been proposed as a strategy to reduce prevalence among PWID—and in turn, lower transmission [7], though questions remain about the actual efficacy of this approach [8]. A systematic review and meta-analysis showed that PWID who receive medically assisted substance use treatment (SA/MAT) and participate in high-coverage syringe access programs may reduce their risk of HCV infection by 70% [9]. This raises the possibility that such strategies could be employed alongside DAA treatment to lower downstream cases of HCV-related severe liver diseases. Previous modeling studies have reported that scaling up MAT, high-coverage syringe access programs, and HCV treatment over time can reduce new infections and disease burden, and advance toward HCV elimination [10]. This paper extends that work by exploring the impact of treating chronically infected PWID at all HCV stages, and describing the anticipated benefits of combining DAA treatment with intensive SA/MAT.

Large-scale studies of combined interventions trials among PWID for either HIV or HCV are rare [11], in large part due to cost and difficulties associated with controlling recruitment and cohort retention over long time scales (as required given the slow impacts of both diseases on health). As a result, the long-term efficacy of combination intervention strategies for HCV is largely unknown. Given the recent rapid rise in opiate abuse, data-driven intervention design is critical to altering the trajectory of HCV infection among PWID. Computer simulation offers the possibility of predicting the long-term dynamics of HCV infection among PWID, and the opportunity to “test” *in silico*, the effect of different combinations of intervention strategies [3]. Where lower-cost SA/MAT interventions can be demonstrated to enhance the impact of DAA treatments, public health officials may be availed of new, more effective strategies to lower HCV prevalence among PWID.

Over the last two decades, researchers have used modeling and simulation to study HIV infection, uncovering important social and behavioral factors related to its prevalence among PWID [12–14]. This prior work has incorporated stochastic population models [15–19] for sexual [20–23] and injection drug co-use networks [24–26], and combinations of both [27, 28]. More recently, analogous approaches have been brought to bear in the context of HCV [29, 30], employing simulations based on both agent-based [31, 32] and networked population perspectives [7, 33]. This ongoing effort emphasizes computational modeling of the projected impact of interventions, considered both singly [7, 34] and in combination [8, 35]. In this paper, we adopt a network-structured, agent-based approach that combines actors with diverse behavioral profiles within the social environment of a dynamic, networked population. Our approach differs from both cohort-based Markov simulations [36] commonly used in cost-effectiveness studies of HCV treatment [37, 38] and the closed population network models found in stochastic actor simulations [29]. The simulations described here model open populations, where agents leave and join the risk population over time, and thus implement more realistic models of the dynamic and mobile PWID communities in which municipalities’ health departments seek to intervene. In addition, we include HIV infection in our models, as serosorting based on HIV status (and HCV status) is known to bias risk partner selection [39], and can be expected to influence the in-network epidemiological dynamics of HCV. Simulation-based research involving open, networked populations is rare, and thus the results presented here represent a significant advance over current HCV and HIV cohort modeling frameworks.

## Methods

### Models

#### System model

At its core, the system is modeled as a dynamic network consisting of nodes (the agents) that are interconnected by a set of edges (their risk-bearing relationships). Nodes are dynamic because they enter and leave the population, and while within the population, they change their relationships, and potentially change with respect to HIV and HCV disease status. The dynamism is regulated and driven by 6 interlinked but autonomous processes that operate within each agent. Collectively, these processes define a dynamic socially networked population in which risk-bearing acts occur and (as a consequence) HIV and HCV propagates. Each individual enters and leaves the population in a manner that is governed by the Initialization and Longevity processes, respectively. Bracketed between these, the node is said to be participating in the risk network, and engages both its Churn and Risk processes. The former (Churn) process alters the local structure of the individual’s risk relationships, while the latter (Risk) process engages current relationships to carry out risk-bearing activity related to drug equipment sharing. Of course, such risk-bearing activity has the potential to cause disease propagation. Each relationship, upon creation, is stochastically assigned an expiration time. Each individual, upon creation, is stochastically assigned both a participation interval, and an ideal risk degree (number of risk partners). The process by which each individual ensures that it always has its ideal number of risk partners (despite disruptions due to Churn and Edge Expiry) is referred to as the Ideal-degree Enforcement process. Further details on interplay of the 6 processes are given in Table 1; an explanation of the parameter values appearing in this Table can be found in the Model Parameters section.

**Table 1:** The six processes operating within each agent.

Process	Description
Initialization (one-time)	When a node is first made, we stochastically determine its properties, including: participation longevity, ideal risk degree, age, race, etc. These assignments are made stochastically, in a manner that is consistent with population-wide univariate distributions (see Table 2). The Ideal-degree Enforcement process is then invoked at the newly created node (see below).
Risk	Each node, every 23.6 days, chooses one of its current set of risk partners uniformly at random, and engages in a mutual risk act. If the pair is HIV-serodiscordant, HIV is transmitted stochastically across the link with probability 4 ⋅ 10^−3^ (resp. 4 ⋅ 10^−5^) if the HIV+ individual is in acute (resp. chronic) state. If the pair is HCV-serodiscordant, HCV is transmitted stochastically with probability 0.009.
Churn	Each node, every 60 days, chooses one of its current set of risk partners uniformly at random, and breaks off the relationship; the Ideal-degree Enforcement process is then invoked at both endpoints of the severed relationship (see below).
Edge expiry	Edges are assigned expiration times when they are created as part of the Ideal-degree Enforcement process (see below). When an edge expiration occurs, we break off the relationship between its two endpoint nodes. The Ideal-degree Enforcement process is then invoked at both endpoints of the severed relationship (see below).
Ideal-degree Enforcement	If a node’s current number of risk partners is lower than the node’s ideal risk degree, we mitigate this by selecting suitably many new risk partners from the subpopulation of other nodes also having a degree below their ideal. This selection is made in a biased manner that is consistent with the bivariate distributions (see Table 3). We create risk edges connecting the node having a degree deficit to each of the selected risk partners, stochastically assigning each new edge an expiration time in the future.
Longevity (one-time)	Nodes are assigned a participation longevity when they are created as part of the Initialization process (see above). When a nodes participation longevity elapses, all its incident edges are prematurely expired, and the node is deleted from the network. Tom maintain constant population size, a new node is made, and its properties stochastically determined via the Initialization process (see above)

#### HCV model

**Fig 1:**
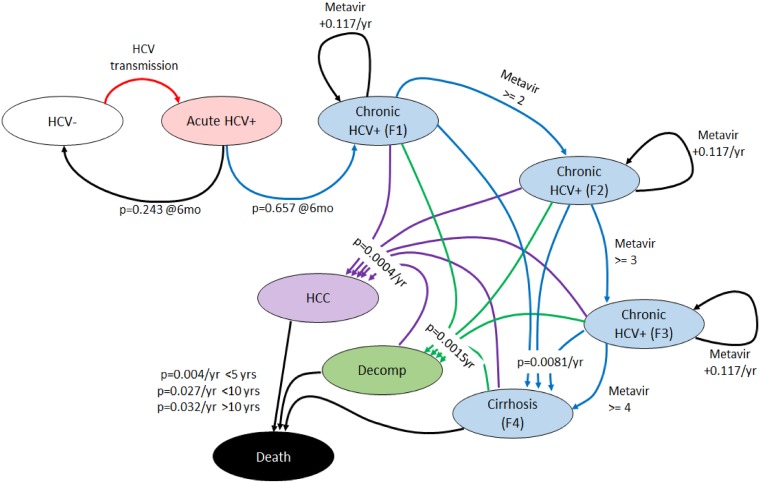
Finite state diagram of the HCV model used in the experiments. Once infected, agents face a series of stochastic and enforced progressions through a series of ever worsening liver function. Throughout the simulation, infected agents who have reached a chronic state (non-acute HCV infected agents) face a small but regular chance of moving directly to cirrhosis, decompensated cirrhosis, or hepatocellular carcinoma. In addition, their Metavir fibrosis level is incremented yearly, moving them gradually from early stage fibrosis to cirrhosis. Once in any of the three severe liver stages, agents face an increasing probability of death due to HCV infection, incremented on a five year basis.

As the main focus of the paper is HCV, the simulation continuously updates the disease status and liver function status of HCV^+^ agents in a manner consistent with the natural history of HCV disease progression (see Fig 1; an explanation of the parameter values appearing in the figure can be found in the Model Parameters section). All uninfected agents who contract HCV through a risk act with an infected alter enter into an acute state. Over the next 6 months of simulation time, they are HCV infected and can transmit the disease to their network alters via risk acts. However, at the end of a six month acute phase, the agent’s status is checked against a probability of spontaneously clearing the infection. The probability of clearance is 0.243. If the agent has not cleared the infection, they enter a chronic HCV state and begin a Metavir fibrosis progression. Here each agent’s fibrosis level is incremented by 0.117 annually. Agents whose cumulative fibrosis level is greater than 1.0 are said to be in Metavir fibrosis level 1; greater than 2 are said to be in Metavir fibrosis 2.0, etc. Agents in Metavir fibrosis level 4 are said to be in *cirrhosis*. Chronically infected agents also have a fixed chance, evaluated annually, of entering into one of three states of severe liver disease: cirrhosis (0.0081), decompensated cirrhosis (0.0015) or hepatocellular carcinoma (0.0004). Once in an advanced liver disease state, an agent faces increasing risk of death each year. HCV pathogen properties of spontaneous clearance were drawn from Smith et al [40], while HCV fibrosis and liver stage transition probabilities (e.g. Metavir fibrosis stage advancement rates and the development of decompensated cirrhosis, and hepatocellular carcinoma) were drawn from published meta-analyses of HCV infection dynamics among PWID [41]. During all HCV infection states, transmission probabilities are set uniformly at 0.009 per risk act.

#### HIV model

**Fig 2:**
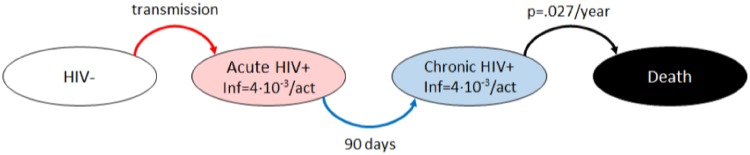
HIV finite state model. Once infected, agents spend 90 days in an acute state with a relatively high probability of transmitting HIV, then pass into a chronic state and lower probability of transmitting an HIV infection. All chronic HIV-infected agents face a yearly stochastic possibility of dying from the infection.

In addition, because HIV infection is possible in the simulation (and HIV serosorting behaviors condition network connections) a model of HIV infection was included in the simulation based on prior work by the research team [24, 25]. Fig 2 describes the natural history of HIV; an explanation of the parameter values appearing in the figure can be found in the Model Parameters section. HIV infection has two stages, and initial acute phase of high infectiousness (probability 4 ⋅ 10^−3^ per risk act), and a chronic phase of low infectiousness (probability 4 ⋅ 10^−5^ per risk act). All infected agents pass through a 90 day acute phase before moving to chronic HIV infection. Chronically infected agents remain in this state until they leave the simulation (at the end of their individual simulation longevity), or die from either HIV or HCV infection. The probability of dying of HIV in the simulation is constant in the chronic state, and is assessed yearly by a stochastic evaluation. As with HCV, these parameters were determined experimentally such that the results stabilized at current levels during simulation burn-in.

### Implementation

Our simulation platform has been described in detail previously [42], and has been used to establish the importance of self-organizing behavioral factors in explaining the non-spreading of HIV among PWID in New York City during the early stages of HIV epidemic [24, 25].

In the experiments described here, the simulation engine begins by creating a population of agents who are assigned individual behavioral and demographic states, disease statuses, risk propensities, and risk network connection tendencies as specified by univariate distributions derived from Centers for Disease Control’s National HIV Behavioral Surveillance (NHBS) project [43], IDU round 3, collected in New York City in 2012 by the New York City Department of Health and Mental Hygiene [44, 45]; see Table 2. Once established as a population, a set of risk relationships are created for each agent—links to network alters across which diseases can be transmitted via singular risk acts. The biases in link formation tendencies are expressed via bivariate distributions (also derived from NHBS data, see Table 3). In the current experiments, these links represent drug co-use relationships with network alters who are regular co-injectors and with whom risk event are most likely to happen. As the simulation begins, agents act independently to perform risk acts with network alters based on their individual risk propensities. They also change network alters through a churn process that periodically removes one of their incident links and replaces it with a new one.

**Table 2:** Per agent univariate parameters in the Baseline settings.

Variable	Source	Value	Percentage
Age	NHBS–“age”	18-24	1.0
		25-34	16.7
		35-44	28.1
		45-54	38.4
		55-65	13.9
Average Degree	NHBS (see below)	0-3	62.1
		4-8	18.4
		9-19	19.5
Connection Duration	Khan et al [42]	Short	40
		Long	60
Gender	NHBS–“gender”	Male	74.9
		Female	24.5
		Transgender	0.1
HCV initial	Smith et al [41]	Negative	50
		Acute	10.0
		Chronic (F0)	30.0
		Fibrosis Stage 1	4.5
		Fibrosis Stage 2	2.0
		Fibrosis Stage 3	1.5
		Cirrhosis	1.0
		Decompensated	0.5
		HCC	0.5
Fibrosis progression	Smith et al [41]	per year rate	0.117
HIV	Neaigus et al [45]	Uninfected	88.0
		Acute	0.2
		Chronic	11.8
Longevity	NHBS–“age-ageinj”	7-30 days	1
		365-7301 days	99
Race/Ethnicity	NHBS–“newrace”	African American	66.7
		Hispanic	13.5
		White	18.8
		Other	1.0
DAA/SVR treatment rate			0.0
Combined SA/MAT treatment rate			0.0

Baseline agent parameter distributions. The degree distribution was the sum of the NHBS variables “num-na; num-ccw; num-dda”. This count provides the number of risk partners—i.e. the expected number of network alters with whom an agent shares needles or other injection related equipment. This number should not be confused with the common measure of RDS network degree often used in data collection with PWID (which is the number of known alters who also inject drugs). The latter is normally much higher than the actual number of risk partners.

**Table 3:** Baseline per agent bivariate mixing parameters.

**Age (binned)**					
	18-24	25-34	35-44	45-54	55-65
18-24	0.0	0.525	0.273	0.202	0.0
25-34	0.083	0.248	0.302	0.255	0.075
35-44	.027	0.184	0.331	0.368	0.091
45-54	0.014	0.110	0.173	0.443	0.174
55-65	0.0	0.087	0.173	0.471	0.269
**Ideal Degree**					
	0-3	4-8	9-19		
0-3	0.518	0.364	0.118		
4-8	0.391	0.417	0.193		
9-19	0.279	0.425	0.296		
**Gender**					
	Male	Female	Trans.		
Male	0.784	0.211	0.005		
Female	0.644	0.345	0.011		
Transgender	0.379	0.288	0.333		
**HCV Status**					
	Positive	Negative			
Positive	0.814	0.186			
Negative	0.286	0.714			
**HIV Status**					
	Positive	Negative			
Positive	0.816	0.184			
Negative	0.144	0.856			
**Race/Ethnicity**					
	Afr. Amer.	Hispanic	White	Other	
African American	0.762	0.100	0.127	0.010	
Hispanic	0.507	0.381	0.112	0.0	
White	0.277	0.048	0.651	0.0	
Other	0.472	0.0	0.528	0.0	

The table should be read as the proportional probability for each variable of forming an outgoing tie from the row to the column.

A *master scheduler* conducts agent actions one at a time in a discrete event framework, such that an action requested by one agent in the population is concluded before the next action of any other agent in the population is begun. To manage this, all actions take place under a single continuous time progression known as the simulation *clock*. The result of each event—such as a change of disease status for one agent that results for a risk act that they are involved in—becomes the starting condition for the next scheduled event by the next agent on the schedule. Throughout the simulation, the outcomes of network churn events, disease transmission events, transitions from chronic to severe liver disorders, and overall agent longevity are determined stochastically, meaning that a random number generated by the simulation is compared with a previously calculated probability distribution to produce an event result. In this way, simulations with identical initial starting points can have different trajectories due different stochastic outcomes. During the simulation, an observer module tracks the current set of network connections and individual disease statuses of every agent, while performing updating functions such as aging the agents according to the progression of the simulation clock, updating HCV disease status as it moves through stages of Metavir fibrosis and on to advanced liver disease, removing agents from the simulation when their participation longevity is reached or when they die as a result of either HIV or HCV disease progression, and creating and introducing new agents into the simulation population to replace those that leave or die. The data sources for both the population and their behavioral tendencies are described below.

A central feature of the simulation approach used here is the inclusion of porous population boundaries. Each agent in the simulation is given a participation longevity—the length of time for which they will remain in the population and be subject to risk events, infection, disease progression and so on. This feature is meant to mimic real world PWID networks where location and co-use relationships are fluid, and which contrasts strongly with population—based study cohorts where retention is assumed. Agent participation longevity is parameterized according to CDC NHBS data (see Table 2), and agents schedule their departure event with the master scheduler when they enter the simulation population. As with real world PWID, this departure can be caused by any number of factors that enforce an end to local participation including entering treatment, incarceration, long-term relocation to a different drug-use network, or death. When an agent leaves the network, the observer module notes the event and creates a new agent whose characteristics are drawn from the original population specifications. In this way the population stays (on average) the same through time, but population turnover takes place in ongoing fashion. As a result, simulations with a consistent population of roughly 10,000 agents over 15 years actually involved the participation of more than 25,000 distinct agent actors. As mentioned above, this approach is meant to lend real-world credibility to intervention scenarios which tend to be local in their implementation and subject to participant mobility/dynamism that necessarily involves a fluid population. We note that the incoming, replacement population remains consistent with the original population parameters which include a mix of new and long-term PWID with infected and uninfected statuses across a range of HCV disease states.

In the current analysis, simulations track long-term HCV infection rates under a range of conditions: 1) Baseline—where no new prevention or treatment activities are involved beyond those already in place in New York City when the baseline data were collected, and where both HCV and HIV percolate through the network based on the results of infections that take place during risk events across agent networks; 2) *prevention*, which includes scaled implementation of MAT and intensified syringe access to reduce pair-wise risk of infection (operationalized as changes in the likelihood of infection taking place in the risk events of those taking part in the intervention); 3) *treatment*, which includes implementation of DAA to chronically infected agents (operationalized as a probabilistic cure of randomly chosen agents in any chronic stage of HCV, i.e. post-acute Metavir fibrosis level zero or above); and 4) combinations of the aforementioned prevention and treatment strategies at a range of implementation scales. Throughout, intervention results are tracked over 15 years from an initial starting point that is based on parameters drawn from PWID surveillance research in New York City in 2012 [45, 46].

The logic of this approach rests on the idea that Baseline simulations represent the likely future of HCV prevalence and incidence over the next 15 years if no *new* actions are taken to prevent infection, and drug use trends and availability remain relatively steady. As such, baseline rates are meant to include current levels of syringe exchange (but not intensive syringe exchange coupled with MAT), anti-retroviral treatment, medically assisted opioid treatment (but not in combination with intensive syringe exchange), and HCV treatment (though not DAAs, which were not available when the data used for the baseline settings were collected). The Intervention scenarios represent possible changes in those Baseline trajectories based on changes brought about through new efforts in prevention or treatment, above and beyond those in place in 2012 when the baseline data were collected. Though a future in which drug use patterns and current intervention and prevention levels remain the same may seem overly idealistic, such an assumption is necessary to isolate the potential impact of simulated prevention and treatment changes.

**Fig 3:**
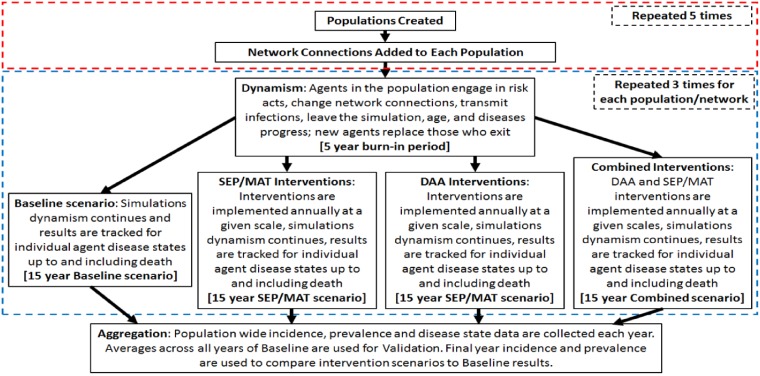
Experiment protocol. For each Baseline or Intervention scenario, 5 artificial PWID populations are created and given initial edge sets. A 5 year simulation burn-in creates independence from the initial population/network starting point and then Baseline or Intervention scenarios are followed. The burn-in and scenario are repeated 3 times.

In the experiments described below, 5 random artificial injection networked populations of 10,000 injectors were created for each of 4 scenarios (Baseline, DAA intervention, SA/MAT intervention, and simultaneous DAA and SA/MAT interventions). For each of the populations, 3 independent simulations were undertaken from the same initial starting point, projecting the population out 15 years into the future after an initial 5 year burn-in (see Fig 3). In the Baseline simulations, the same initial settings guided the entire simulation as actors followed their behavioral characteristics for risk and network churn, and HCV and HIV percolated throughout the population. In the Intervention settings, key agent parameters were altered to mimic the effects of an intervention. This took place immediately following the burn-in period, and were repeated each year according to the intervention descriptions below.

The overall population levels for the simulation (i.e. 10,000 agents) is meant to reflect a single, open region within a larger urban zone. Such geographical concentrations are well known in most urban areas and represent permeable spatial and social foci where individual PWID are associated for varying lengths of time [47]. The burn-in period for the simulation is meant to produce a simulation environment that is free from initial starting conditions. Generally, HCV prevalence during the burn in period rises as HCV is transmitted across the risk edges to uninfected agents. In the Baseline scenarios, HCV (and HIV) prevalence and incidence remain relatively static throughout the next 15 years, despite the fact that agents come and go from the network and new infections continue to take place. In the Intervention scenarios, changes to agent behaviors (prevention) or disease states (treatment) alter the incidence and prevalence trajectories away from the Baseline settings.

Over the course of each scenario, agents commit risk acts across their existing network relationships, and form new relationships whenever their existing connections dissolve. Agents also leave the simulation population when their participation longevity elapses. Whenever an agent leaves the simulation, his/her network connections are dissolved and his/her HCV infection status is removed from future measurements. To replace departing agents, new agents are created and given their own risk partnerships with current members of the population. Arriving agents continue to have a 50% chance of being HCV infected at inception (as per initial conditions—simulating a situation where a mixture of new injectors and those who have been injecting for some time enter the population from the surrounding area). At each step of the simulation, population-wide prevalence is measured by considering the status of only those agents who are still in the simulation. This modeling paradigm reflects real-world conditions where community boundary conditions are fluid and populations are mobile: PWID change locations, enter addiction treatment, become incarcerated, and so on, all of which removes them from their risk networks, while other, previously unknown PWID take their place in an evolving network milieu (54). One result of this is that incarceration rates, cessation rates, death rates due to non-HIV or HCV causes, and relocation out of the imagined study area are not treated as separate parameters. Instead, they are subsumed under the single longevity variable that simply assigns a participation duration to every agent—leaving the specific cause of their exit undetermined. Individual infections of both HIV and HCV also age as the simulation progresses. Metavir stages are incremented for those infected with HCV, and stochastic opportunities for sever liver disease progression are made each year (with probabilities set to match known population level outcomes). Among the possible outcomes is death via liver-related disease [48].

### Model parameters

Data for simulation populations are drawn from the Centers for Disease Control’s National HIV Behavioral Surveillance project [43], IDU round 3, collected in New York City in 2012 by the New York City Department of Health and Mental Hygiene [44, 45]. Table 2 provides the univariate distributions drawn from participants who were verified to have injected any drug in the past year at the time of their interview. These data were used to create each individual agent’s age, gender, average network degree, race, and longevity of participation within the simulation population. All variables are treated as categorical to enable discrete mixing probabilities. Where ranges are present within a category (i.e. “age” or “average degree”), agents are assigned a random value within the range.

Mixing patterns between agents were determined by aggregating the agent x agent bivariate probabilities across all categories to create a relatively likelihood of connection from one agent to another. Stochastic throws against relative likelihoods were used to determine actual agent connections both initially and in dynamic fashion as the simulation progresses. Table 3 quantifies the homophily biases on population mixing (by age, average degree—binned, gender, HCV status, HIV status, and race). To remain true to the original recruitment data, these distributions often reflect asymmetrical mixing tendencies common to PWID—meaning that the tendency of individuals in group A to seek partners from group B may not be the same as the tendencies of B’s to seek partners from A. The parameters in these tables were drawn from both the NHBS interview data and the respondent driven sampling recruitment data, analyzed and weighted via RDSAT [49]. Tendencies toward serosorting behaviors for both HIV and HCV infection are drawn from published sources [39, 50, 51]. Prior research has shown that serosorting condition the likelihood of pairwise risk relationships [52], and the use of serosorting data has yielded conclusions which are consistent with what is known about the long-term behaviors of the PWID population [25] in New York City. Distributions quantifying the duration of risk relationships were drawn from consultation with ethnographers working in the area and prior research with PWID in New York City [25].

Risk acts are simulation events where an agent, according to their own risk tendencies, decides to engage in an act with a current network alter that can potentially transmit HCV or HIV. These can be thought of needle or equipment sharing events between two people already in a drug co-use relationship. The frequencies were drawn from the interview portion of the CDC’s NHBS survey that deal with injection risk: NHBS researchers asked the frequency with with respondents inject drugs (*injavr*), and the rate at which the respondent had used injection related paraphernalia after someone else had used it in the preceding 12 months, including needles (*sharndle*); cookers (*sharcookr*); cotton (*sharcottr*); water (*sharwatrr*); works (*sharworkr*); used a previously used syringe to divide drugs, i.e. “backloading” (*samessyrr*). Because these measures were collected along a qualitative scale, they were recoded as numbers that reflected ranked intervals (0 = never, 1 = rarely, 2 = half time, 3 = most time, 4 = always). The individual values were summed for each respondent, and then divided by the reported injection frequency. This created a distribution of relative risk rates across the NHBS population that, while lacking an absolute scale, differentiate population according to their frequency of engaging in risky injection practices with their network alters. Preliminary simulation experiments were used to scale this relative parameter to an absolute number of days such that risk rates converged on the HCV and HIV prevalence levels obtained by the CDC NHBS study over the burn-in period, and remained near these levels during Baseline simulations (see “Validation” below). At their creation, agents are randomly assigned an absolute risk rate within this “pegged” distribution. The result is that the average agent engages in one risk act capable of transmitting HIV or HCV every 23.6 days.

As above, per-risk-act infection probabilities for HCV were also treated as a tuning parameter, and were fitted to the NHBS data along side the absolute risk rates discussed above. The final setting (0.009 chance of infection per risk act across HCV discordant pairs in an active risk relationship) produced prevalence levels (60-70%) that match current HCV levels in the NHBS New York PWID population (and among US PWID more generally [1, 45]—see Boelen et al [53] for a discussion of defining transmission probability via equipment sharing).

### Validation

To validate the Baseline settings, 15 year simulations were performed using the cross-section data from the NYC NHBS as coded in settings shown in Tables 2 and 3 and the HIV/HCV models described above (i.e. the “Baseline scenario”), and the results compared to historical trend data from NYC [45, 46]. The goal of the validation experiments was to show that the Baseline settings would cause the simulation to converge at a relatively steady state that matched the known population incidence and prevalence levels of the PWID population in New York City. Where simulations converge and maintain current levels of HCV and HIV risk in dyanamic fashion, this indicates that ongoing risk acts, network churn, and population turn over result in a dynamic equilibrium in line with what is know about historical HCV and HIV trends from the same area. As such, these results show that the simulation produces known macro-level results as *emergent phenomena* based only on the actions of the agents themselves. Given such a dynamic equilibrium, comparisons can be made between the relatively steady state Baseline and later Intervention scenarios in which one or more Baseline parameter is changed [54]. In addition, the low level of variation seen in the simulation trajectories show that despite the stochastic nature of the system, 15 simulations were sufficient to capture the overall effects (see standard deviations across 15 trials in Table 4).

**Table 4:** Average incidence and prevalence of HCV states in Baseline simulations.

HCV State	Avg Incidence (std)	Published Rate	Avg Prevalence (std)	Published Rate
Uninfected				33.9 (2.1)
Acute	11.841 (.420)		4.6 (.2)	
CC	0.599 (.023)	0.662 [46]	3.9 (.5)	
DC	0.088 (.006)	0.182 [46]	0.8 (.1)	
HCC	0.024 (.002)	0.032 [46]	0.5 (.1)	
All HCV States			66.1 (1.8)	67.1 [45]

To validate the Baseline settings, incidence and prevalence rates were tracked across 15 years of simulation time (following a 5-year burn-in period). Means across 15 independent simulations of networked populations of 10,000 actors for all years of each simulation are here compared with published results. Acute incidence is expressed per 100 person years of all uninfected agents. Cirrhosis (CC), decompensated cirrhosis (DC), and hepatocellular carcinoma (HCC) incidence is expressed per 100 person years of all HCV infected agents.

For these validation runs (and for all subsequent experiments) the population-wide initial prevalence of HCV infection prior to the burn-in period was set at 50%, meaning that during the population creation process, each agent is given a 50% chance of being assigned an HCV infection state (according to a distribution of liver disease statuses drawn from published sources, see Table 2). During 5 years of simulation “burn in” (used in both the baseline and intervention scenarios) HCV prevalence stabilizes at 60-65%, and HIV prevalence stabilizes at 12-15%. Such conditions are meant to approximate current conditions in New York City [45], and generally reflect PWID HCV prevalence rates in urban areas through the US at the current time.

The long-term results of the validation simulations are shown as averages across 15 years of simulation after the 5 year burn-in period at the Baseline settings, across 15 independent simulations (for a total of 225 data points). Robustness and sensitivity tests for these results are discussed in the Limitations section below. Validation of the Baseline model can be seen in the incidence (per 100 person years) and prevalence of the main outcome variables: acute HCV infection, overall HCV infection rate, rates of cirrhosis (CC), decompensated cirrhosis (DC), and hepatocellular carcinoma (HCC) (see Table 4). These results can be compared to those of recent meta-analyses [41] and NYC incidence and prevalence estimates [45, 46]. The only major difference noted in the validation outcomes is in the rate of decompensated cirrhosis (0.088 simulation average versus reported rates of 0.182 [46]). For consistency, the disease state transition probabilities were drawn from a meta-analysis by members of our team using data from 1990-2013 [41], while the comparison statistic cites rates from 2006 to 2013 [46]. As such, the discrepancy may reflect changes in the nature of the HCV epidemic over time. For comparison, we note that one of the only prospective studies of HCV progression among PWID [55] found high variance in progression rates to severe liver disease, including jumps to Fibrosis stage 5 or 6 from much lower prior Fibrosis levels (including levels 1 and 2). Because all final rates in the simulation are emergent phenomena that are dependent on the number of agents in a given state of Fibrosis, exact fitting of every state turned out to be problematic. Where the rates are comparable, we proceed with the progression figures suggested by Smith et al [41], but note that the final simulation results for decompensated cirrhosis are likely lower than suggested by current data.

### Interventions

In the experiments described here, we test the effect of a range of intervention scenarios:

- A Baseline scenario where neither of the interventions described below take place. In the Baseline scenario there is no direct acting antiviral HCV treatment (such treatments were unavailable at the time, though 34% of NHBS NYC respondents reported having received some medication for HCV infection ever in their lifetime, and 29 of 526 reported having been cured of HCV in the past). Syringe exchange services were active in the NHBS sample (88% of NHBS respondents indicated that they had receive at least one syringe for a such a service in the last year), but coupled intensive syringe exchange where all needles are received from an exchange and simultaneous medically assisted opiate addiction treatment were not available (see [3] for a similar distinction). Specific data on ongoing medically assisted opioid treatment is not available for the the 2012 NHBS NYC data, though 63% of those interviewed reported some participation in drug treatment in the last 12 months. Because the intervention scenarios discussed here go well beyond simple syringe access or prior non-DAA HCV treatments, for the purposes of the current experiments, the level DAA (and corresponding “sustained virological response” (SVR) and combined intensive SA/MAT in the Baseline scenarios are set to zero. We note, however, that evidence of a willingness to engage treatment and the potential for syringe services to reach large proportions of the population are clear from these data.
- A DAA intervention implemented on the first day of year 6 (following the 5-year burn in period) and on the first day of each subsequent year. Here a fixed percentage of the current, post—acute, chronically infected HCV population is randomly selected for participation in the intervention, and their intervention start date is randomly assigned to occur in the next 365 days. Selection is made across all Metavir fibrosis states F0 through cirrhosis (F4). During the intervention, participants undergo DAA treatment while continuing to practice drug use and incurring risk of HIV infection, but the odds of contracting HCV during this period is zero. Reflecting research findings, 90% of those agents adhere to HCV DAA treatment for the entire duration (168 days/24 weeks), and 95% of those who complete it are cured [56]. At the end of treatment adherence period agents are placed back into the simulation population and continue to operate in a manner specified by their individual agent parameters. During the post-treatment period, reinfection with HCV is possible for as long as the agent remains in the simulation. This is determined stochastically via risk acts in the same way that the initial infection was created. In the case of reinfection, Metavir fibrosis levels resume at the level reached prior to treatment and once again begin to progress toward cirrhosis. Other than treatment-based changes in disease status, at the micro-scale, DAA participants operate in a manner consistent with Baseline.
- Intensive syringe access and opiate substitution treatment (SA/MAT), at various levels of recruitment/participation. Here, in year 6 and in each year following, a fixed percentage of the total population in the simulation is recruited for participation regardless of HCV status. During the year that follows, each participating agent’s number of risk acts with their network neighbors is decreased by 80% from its original pre-intervention level [57]. At the end of the intervention period, participating agents return to their pre-intervention risk act rate. Because the selection of participants is random each year, individuals may be re-recruited in subsequent years (or even two or more years in a row), depending on the luck of the draw; or they may leave the intervention prior to reaching the 365 day intervention duration, depending on their likelihood of completing the program (determined stochastically). The result is a distribution of participation that may last from a few months to several years in the intervention. In cases where a high percentage of the population is enrolled each year, longer periods of continuous reduced risk becomes more likely. Other than intervention-based changes in risk behavior, at the micro-scale, SA/MAT participants operate in a manner consistent with Baseline.
- To test the effect of combination strategies, additional scenarios were considered where both the DAA treatment and SA/MAT participation were engaged concurrently (and independently) in the same simulation population. The rationale for this set of scenarios was to determine whether and to what extent each of these approaches—direct-acting antivirals and opiate substitution/syringe access—might, in combination, further reduce levels of HCV in the population compared against approaches relying on each strategy taken alone, and against a Baseline matching conditions akin to those in 2012.

## Results

The final prevalence rates for the 15 years after burn in for Baseline and Intervention implementation (for a range of DAA and SA/MAT interventions scales) are found in Table 5. The long-term effects of combined interventions at various scales are considered next, including measurements of chronic prevalence (Table 6), prevalence of HCV-related advanced liver disease (CC+DC+HCC; see Table 7), HCV incidence (Table 8), and incidence of cirrhosis (Table 9).

**Table 5:** Population proportion by HCV disease state after 15 years of intervention: Mean (std).

Intervention	Uninfected	Acutely infected	F0-3	CC+DC+HCC	Total
Baseline	.384 (.005)	.049 (.002)	.527 (.005)	.041 (.002)	.616
DAA 5%	.440 (.004)	.051 (.002)	.475 (.004)	.035 (.002)	.560
DAA 10%	.491 (.006)	.051 (.003)	.427 (.005)	.031 (.002)	.509
DAA 15%	.535 (.004)	.052 (.002)	.390 (.004)	.027 (.002)	.465
DAA 20%	.574 (.004)	.049 (.002)	.352 (.004)	.024 (.001)	.426
SA/MAT 20%	.400 (.008)	.045 (.003)	.514 (.007)	.041 (.002)	.600
SA/MAT 40%	.417 (.005)	.040 (.002)	.503 (.005)	.040 (.002)	.583
SA/MAT 60%	.438 (.005)	.034 (.002)	.489 (.006)	.039 (.002)	.562
SA/MAT 80%	.457 (.005)	.028 (.002)	.475 (.006)	.040 (.002)	.543

Measures after the completion of 15 years simulation that followed a 5-year burn-in period. Results show the mean and standard deviation for each state from 15 independent simulations of networked populations of 10,000 actors.

**Table 6:** Population proportion of Chronic (F0-F3) after 15 years of combined interventions.

Intervention	Baseline	DAA 5%	DAA 10%	DAA 15%	DAA 20%
Baseline	.527 (.005)	.475 (.004)	.427 (.005)	.390 (.004)	.352 (.004)
SA/MAT 20%	.514 (.007)	.477 (.006)	.413 (.003)	.377 (.004)	.343 (.004)
SA/MAT 40%	.503 (.005)	.453 (.007)	.403 (.004)	.366 (.005)	.332 (.004)
SA/MAT 60%	.489 (.006)	.436 (.006	.391 (.005)	.355 (.004)	.323 (.005)
SA/MAT 80%	.475 (.006)	.423 (.006)	.378 (.005)	.345 (.004)	.313 (.004)

Measures after the completion of 15 years simulation that followed a 5-year burn-in period. Results show the mean and standard deviation for each state from 15 independent simulations of networked populations of 10,000 actors. Acute infections are not included in this table to avoid counting those who spontaneous clear the infection during the simulation year.

**Table 7:** Population proportion of HCV-related advanced liver disease (CC, DC, HCC) after 15 years of combined interventions.

Intervention	Baseline	DAA 5%	DAA 10%	DAA 15%	DAA 20%
Baseline	.041 (.002)	.035 (.002)	.031 (.002)	.027 (.002)	.024 (.001)
SA/MAT 20%	.041 (.002)	.034 (.002)	.031 (.001)	.027 (.002)	.023 (.001)
SA/MAT 40%	.040 (.002)	.035 (.003)	.030 (.002)	.026 (.002)	.023 (.002)
SA/MAT 60%	.039 (.002)	.033 (.002)	.029 (.001)	.025 (.001)	.023 (.001)
SA/MAT 80%	.040 (.002)	.034 (.002)	.030 (.002)	.025 (.002)	.023 (.002)

Measures after the completion of 15 years simulation that followed a 5-year burn-in period. Results show the mean and standard deviation for each state from 15 independent simulations of networked populations of 10,000 actors.

**Table 8:** Incidence rate of HCV among uninfected agents (in 100 person years).

Intervention	Baseline	DAA 5%	DAA 10%	DAA 15%	DAA 20%
Baseline	11.00 (0.25)	10.40 (0.35)	9.37 (0.39)	8.84 (0.28)	7.89 (0.33)
SA/MAT 20%	9.43 (0.68)	8.72 (0.30)	8.07 (0.28)	7.35 (0.16)	6.74 (0.32)
SA/MAT 40%	7.74 (0.37)	7.44 (0.67)	6.33 (0.33)	5.90 (0.21)	5.29 (0.26)
SA/MAT 60%	6.02 (0.38)	5.58 (0.41)	5.02 (0.32)	4.46 (0.28)	4.16 (0.28)
SA/MAT 80%	4.38 (0.29)	4.16 (0.30)	3.57 (0.28)	3.23 (0.20)	2.93 (0.15)

Measures after the completion of 15 years simulation that followed a 5-year burn-in period. Results show the mean and standard deviation for each state from 15 independent simulations of networked populations of 10,000 actors.

**Table 9:** Incidence rate of Cirrhosis among all infected HCV agents (in 100 person years).

Intervention	Baseline	DAA 5%	DAA 10%	DAA 15%	DAA 20%
Baseline	.554 (.040)	.531 (.076)	.500 (.060)	.450 (.067)	.405 (.065)
SA/MAT 20%	.570 (.099)	.521 (.081)	.474 (.044)	.435 (.070)	.390 (.062)
SA/MAT 40%	.521 (.053)	.512 (.070)	.473 (.059)	.406 (.076)	.370 (.068)
SA/MAT 60%	.552 (.047)	.473 (.072	.432 (.057)	.399 (.079)	.385 (.050)
SA/MAT 80%	.515 (.063)	.479 (.066)	.435 (.065)	.381 (.054)	.339 (.057)

Measures after the completion of 15 years simulation that followed a 5-year burn-in period. Results show the mean and standard deviation for each state from 15 independent simulations of networked populations of 10,000 actors.

The tabulated results show that the effects of SA/MAT treatment marginally enhance the effectiveness of DAA treatment in lowering chronic HCV prevalence in an open population (decline of 7%), but have little effect on HCV-related advance liver disease (decline of 1%), with roughly linear effects due to scale for HCV prevalence. However, a different pattern emerges when we turn our attention to incidence rates. Here we see that SA/MAT interventions have significant impacts. As seen in Table 8, high levels of SA/MAT recruitment (at 80% treatment level) have the effect of lowering overall HCV incidence by more than 60%, while scaled DAA treatment at even the highest levels tested here produces a less significant effect (a decrease of 28%). Even at low intervention levels (20%), SA/MAT treatment lowers HCV incidence by 21-39% when combined with DAA treatments of 5% to 20%, with the impact increasing as DAA intervention levels rise. SA/MAT interventions have less of an impact on cirrhosis incidence associated with HCV infection, but still serve to lower cirrhosis incidence by 6-39% when combined with DAA treatments from 5% to 20%. As seen in Table 9, these impacts continue to grow as SA/MAT increases in scale of coverage.

**Fig 4:**
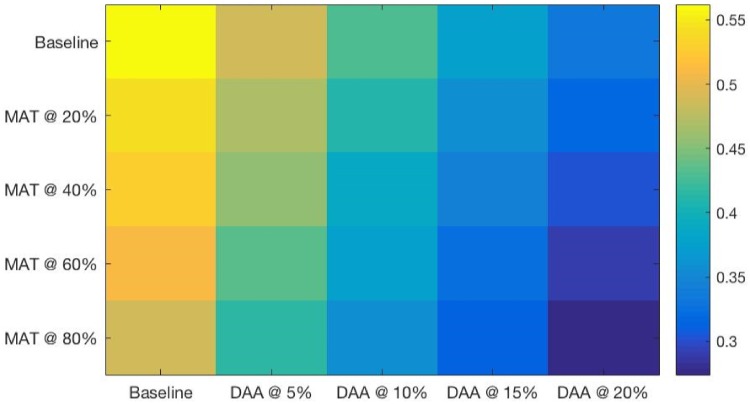
Chronic HCV (F0-3) prevalence at a range of combined intervention scales after 15 years of simulated intervention. Thresholds can be seen at the DAA 20% scale for all levels of SA/MAT.

**Fig 5:**
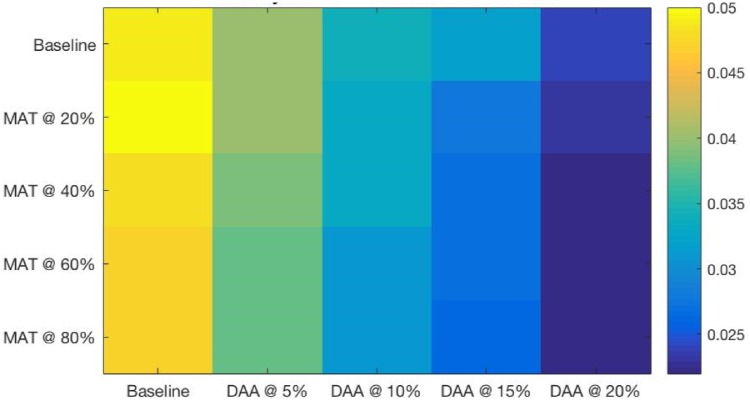
Advanced HCV-related liver disease prevalence at a range of combined intervention scales after 15 years of simulated intervention. Strong thresholds can be seen at the DAA 20% scale for all levels of SA/MAT.

The heatmaps shown in Figs 4 through 7 demonstrate both continuous and thresholding behaviors in the combined interventions. Chronic HCV prevalence (Fig 4) and the prevalence of advanced liver diseases (Fig 5) show continuous declines as DAA levels increase, with relatively fewer (but still continuous) declines provided by increased levels of SA/MAT.

**Fig 6:**
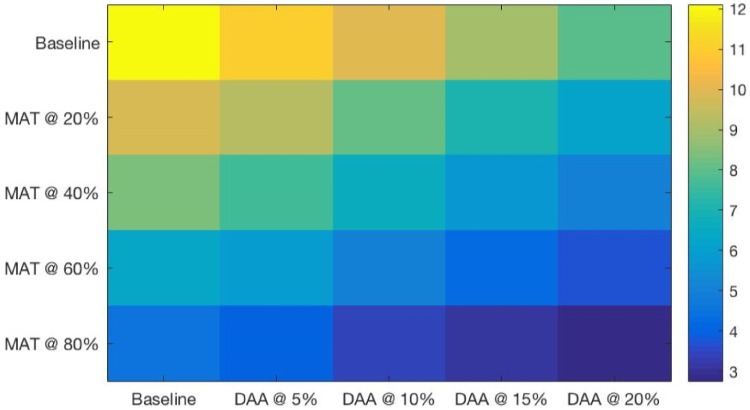
HCV incidence (in 100 person years) at a range of combined intervention scales after 15 years of simulated intervention. Strong thresholds can be seen at the SA/MAT 80% scale for all levels of DAA treatment, and for SA/MAT 60% at DAA treatments of 10% or higher.

**Fig 7:**
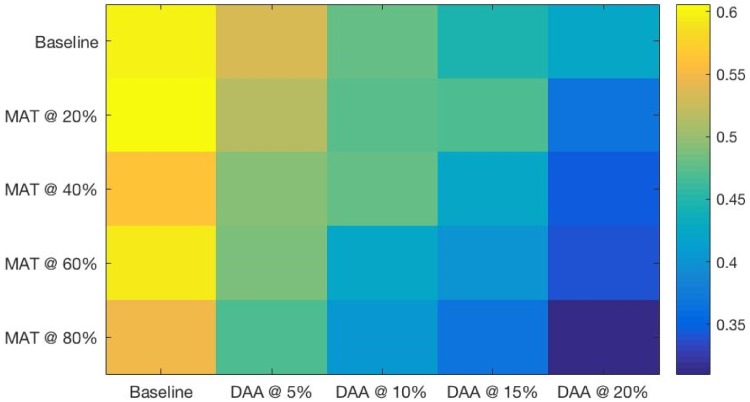
Cirrhosis incidence at a range of combined intervention scales after 15 years of simulated intervention. A notable threshold can be seen at the DAA 20% and SA/MAT 20% scale. There the inclusion of even low levels of SA/MAT can combine with higher levels of DAA to produce considerable effects on cirrhosis incidence in later years.

## Discussion

This simulation experiment represents an attempt to model open populations of PWID over relatively long time periods, and to test the effectiveness of single and combined interventions under such conditions. The scenario we envision is quite common to municipalities and public health departments facing rising HCV rates associated with increases in injection drug use: what are the best strategies for reducing HCV incidence and prevalence and subsequent severe liver related disorders? Given the high mobility of PWID and the long time periods needed for intervention impacts to manifest, intervention planners need to know what can be expected of new disease containment strategies based on the administration of direct acting antivirals, the deployment of medically-assisted addiction treatment and high-coverage syringe access programs, and combinations thereof.

The open nature of the simulations added considerable dynamism to a model that already included regular network “churn”—the effect of agents leaving some risk relationships and entering into relationships with new alters. Despite the fact that the simulation population was held steady at a level of 10,000 agents, in the combined 5 years of burn-in and 15 years of simulation more than 28,000 agents participated in the simulation (on average); more than 330 thousand risk relationships were made and unmade; more than 131,000 intervention events took place; and approximately 26,000 total new infections (HIV and HCV) occurred. The effects of overall HCV incidence and prevalence we report here were thus consistent even amid considerable network change and population turnover.

The final prevalence rates shown in Table 5 show similarities to previous results from Europe [3]. In particular, SA/MAT shows limited ability to lower overall HCV prevalence (declines of 2-7%) across all HCV states—and very little detectable effect (roughly 1%) on advanced liver disease after 15 years [58]. This is not surprising, given that SA/MAT does not cure infection—and thus can not affect prevalence rates directly. However, as reflected in the declining prevalence of acutely infected agents (which show a 42% decrease from baseline levels of roughly 5% to under 3%), SA/MAT can be expected to result in a steady decline in overall HCV incidence that is not captured in the timescale investigated here.

Scaled DAA treatment shows the ability to lower HCV rates directly, resulting in a more than 30% decline when 20% of those with chronic HCV infection are treated each year. In contrast to SA/MAT intervention, there is very little effect on incidence of acute infection. The simulations also show a large drop in the prevalence of advanced liver disease associated with DAA treatment over the 15-year intervention period (reduction of more than 40%). These results were obtained despite the unbounded nature of the population and the fact that many of those who undergo DAA treatment subsequently leave the simulation in the future and are replaced by others who did not undergo treatment, and occurs despite the fact that reinfection after treatment remains a possibility for agents throughout their time in the simulation.

The results of the simulations also verify previous research findings that SA/MAT interventions, acting alone, do little to reduce overall prevalence of HCV infection or the related advanced liver diseases [59]. However, our result show that SA/MAT does have an important effect on HCV *incidence* (including a decline of of 60% when 80% of the agents are enrolled)—an effect that, when coupled with even low levels of DAA treatment, can magnify the much smaller declines in HCV incidence under DAA treatment alone (a decline of 28% at the highest intervention level tested here, where DAA treatment was carried out for 20% of the infected agents each year). In the real world, the long term effects of this are shrouded by the very long time scales associated with advancing HCV, and by the open boundaries on the simulation community—whereby the effects of treatment on the current agents are lost when those treated leave the population. Despite this, even low rates of DAA (5% annually) can be coupled with moderate SA/MAT (40%) to produce a 32% drop in HCV incidence and a 8% drop in cirrhosis incidence over 15 years. In contrast, the same DAA treatment that takes place without accompanying SA/MAT shows smaller declines in HCV incidence (4%), ensuring that treatment needs remain high in the future. High levels of DAA (20%) and SA/MAT (80%) in combination show an ability to lower both HCV (resp. cirrhosis) incidence by more than 73% (resp. 39%), when compared to baseline levels. Importantly, these effects can be anticipated despite high population turnover, and take place amid a steep drop in the overall prevalence of chronic HCV (decline of 21%).

What is apparent from these simulations is that deployment level influences the impact of interventions in a non-linear manner. 5% increments in the number of agents enrolled in DAA treatment per year reduce HCV prevalence on average by 5.2, 4.8, 3.7, and 3.8% respectively, as DAA treatment rates go from 5 to 20% of chronically HCV infected agents (Table 6). As above, SA/MAT show little impact on prevalence, such that increases or decreases in marginal utility are not found. On the other hand, when looking at HCV incidence, accelerating marginal gains can be seen in SA/MAT increments of 20% result which result in relative declines in HCV incidence of 14, 18, 22 and 27%. The impact of increasing DAA coverage is mixed: decreases in HCV incidence of 5.5, 9.9, 5.7 and 10.7% can be seen as DAA coverage is increased by 5 to 20% in 5% increments. Increasing both of these interventions simultaneously results in similar non-linear gains of 21, 27, 30, and 35% relative to each prior increment. However, these results show that combining of DAA and SA/MAT interventions is linear mechanism the total gain is the sum of the impacts of each in isolation.

One can see in the heatmaps limited but important threshold behaviors in the combined DAA and SA/MAT results (see Figs 4 through 7). For example, for HCV incidence (Fig 6), increasing DAA coverage from zero coverage to 5% coverage while holding SA/MAT rates at 40% results in a relative decrease of HCV incidence of 14.9% (where the average gain for increasing DAA by one step across all SA/MAT levels is an 8.6% decrease in HCV incidence). Similarly, when holding DAA treatment rates steady at 5%, increasing SA/MAT enrollment from 40 to 60% results in a 25% relative decrease in HCV incidence. For cirrhosis incidence (Fig 7, a jump from 40 to 60% SA/MAT coverage results in an 8.7% decrease (average gain for SA/MAT increase of 5% is 3.1%), while an increase of DAA treatment from 10 to 15% of the infected population while holding SA/MAT rates steady at 40% resulted in a 14.1% decrease in cirrhosis incidence after 15 years. The largest incremental decrease in cirrhosis incidence resulted from adding 5% DAA coverage to 60% SA/MAT coverage (a drop of 14.3%, compared to an average marginal gain of 8.6% for each 5% increase in DAA coverage). Such discontinuous gains offer health agencies opportunities to assess how best to increase their overall effectiveness where financial or other means prevent them from capitalizing on the accelerating marginal gains seen in increasing both interventions simultaneously.

### Limitations

The most significant limitation on these results is the obvious fact that these findings were obtained via simulation, and thus ignore a range of possible influences external to the simulation environment—such as future changes in drug markets and drug availability, the impact of possible historical events (such as the long term implications of the unfolding opioid epidemic in the US), changes in PWID routes of administration [60], or new local efforts aimed at changing risk behaviors. Though our framework attempts to engage more realistic intervention conditions through the simulation of an open, networked population, possible errors in these data and their basis in New York City also limit their possible extension to other locations.

To test the robustness of our results against possible sensitivity issues unanticipated in the modeling framework, we conducted sensitivity tests that examined significant changes in input parameters closely connected to HCV spreading in the population. Using “one-factor-at-a-time” strategies [61], we perturbed 5 key parameters by ±15% from initial levels: (i) the network churn rate, (ii) the risk rate, (iii) the HCV transmission probabilities, and initial prevalence levels of both (iv) HIV and (v) HCV (see Table 10). We note that, following Best Practice VI-6 of the ISPOR guideline [62], this table is intended to present data on experiment sensitivity (the effect on outputs of varying inputs) and is not intended to speak to questions of uncertainty. The latter type of analysis would require formal determination of parameter uncertainty—a task which is beyond the scope of this paper, given the present state of the literature. Here we take an “even if” approach (see [62], pp. 838) that identifies a range of parameter values that do not change our conclusions.

**Table 10:** Sensitivity test showing the effects of parameter changes of 15% on HCV prevalence under both baseline and intervention conditions.

	Baseline	(orig. 0.616)	DAA 10/MAT 40	(orig. 0.473)
	+15%	-15%	+15%	-15%
(i) Churn rate	0.613 (0.005)	0.618 (0.005)	0.467 (0.004)	0.476 (0.010)
(ii) Risk interval mean	0.590 (0.007)	0.630 (0.008)	0.445 (0.005)	0.4814 (0.002)
(iii) Transmission probability	0.638 (0.006)	0.596 (0.008)	0.483 (0.007)	0.467 (0.011)
(iv) Initial HIV prev.	0.617 (0.005)	0.615 (0.006)	0.475 (0.008)	0.478 (0.003)
(v) Initial HCV prev.	0.671 (0.002)	0.570 (0.006)	0.532 (0.006)	0.420 (0.004)

Measures after the completion of 15 years simulation that followed a 5-year burn-in period. Results show the mean and standard deviation for each state from 5 independent simulations of networked populations of 10,000 actors.

Comparing intervention and baseline scenarios, the data in Table 10 shows that parameter perturbations had minimal impact on the reduction in HCV prevalence after 15 years. More precisely, DAA10/MAT40 intervention reduces HCV prevalence to between 75-77% of its initial value over 15 years, regardless of perturbations of ±15% in parameters (i)-(iv); a fluctuation of 30% in one of the parameter values (i)-(iv) is thus seen to produce < 2% fluctuation in the intervention’s impact on HCV prevalence after 15 years. Sensitivity to initial HCV prevalence is greater; here we found that perturbations of ±15% in parameter (v) produced ∼ 6% fluctuation in the reduction HCV prevalence (73-79% of the initial value). The heightened sensitivity of final HCV prevalence to initial prevalence levels is not surprising given the open population model and the fact that new agents entering the simulation take their HCV status probabilistically from the initial settings. In effect, the overall impact of changes in simulation parameters is dampened by the simulation process itself. From these tests, we surmise that our results are robust against errors of ∼ 15% in initial settings, and that the results are unlikely to reflect hidden sensitivity effects.

Further limitations include recognized elements of HCV progression that were not included in the simulation such as HIV co-infection impacts on HCV progression (which can increase Metavir progression rates by as much as 50% [63], though this impact is lessened considerably by HIV patients receiving ARV treatments [64]). In the simulation, all HCV infected agents advance at the same Metavir fibrosis rate. We also note that new DAA treatments have shortened the treatment period from 168 days (24 weeks) for roughly half that time [65]. Preliminary tests indicated that this does not significantly impact the results presented here, but future work will include a range of DAA treatment times.

## Conclusion

These results suggest significant and immediate steps for health officials and harm reduction programs. Combined interventions that match lower-cost syringe access and medicine assisted addiction treatments with direct acting antiviral treatments can have a large effect on HCV incidence, cirrhosis incidence, and overall HCV prevalence, even among highly mobile PWID populations. Simulations suggest that lower cost syringe exchange and medically assisted opioid treatment can be combined with less available direct acting antiviral treatments to radically lower HCV incidence among people who inject drugs, even when that population is highly mobile and where treatment is at times short term and only temporarily successful. These outcomes are contingent on opening up participation in direct acting antiviral treatment to drug users prior to their entry into later stage fibrosis, and on the ability of health agencies to sustain intervention efforts for more than a decade.
